# A 2-year longitudinal follow-up of performance characteristics in Chinese male elite youth athletes from swimming and racket sports

**DOI:** 10.1371/journal.pone.0239155

**Published:** 2020-10-12

**Authors:** Kewei Zhao, Andreas Hohmann, Irene Faber, Yu Chang, Binghong Gao

**Affiliations:** 1 School of Kinesiology, Shanghai University of Sport, Shanghai, China; 2 Shandong Sport University, Jinan, China; 3 China Institute of Sport Science, Beijing, China; 4 Institute of Sports Science, University of Bayreuth, Bayreuth, Germany; 5 Institute of Sports Science, University of Oldenburg, Oldenburg, Germany; 6 Shanghai Sports School, Shanghai, China; 7 School of Physical Education and Sport Training, Shanghai University of Sport, Shanghai, China; University of Illinois at Urbana-Champaign, UNITED STATES

## Abstract

Training in elite sport aims at the optimization of the athletic performance, and to control the athletes`progress in physiological, anthropometrical and motor performance prerequisites. However, in most sports, the value of longitudinal testing is unclear. This study evaluates the longitudinal development and the influence of intense training over 2-years on specific physiological performance prerequisites, as well as certain body dimensions and motor abilities in elite youth athletes. Recruited between 11–13 years of age at Shanghai Elite Sport school, the sample of student-athletes (N = 21) was categorized as the swimming group (10 athletes), and the racket sports group (11 players: 7 table tennis and 4 badminton players). The performance monitoring took place over two years between September 2016 and September 2018 and included 5 test waves. In all the test waves, the athletes were assessed by means of three physiological measurements (vital capacity, hemoglobin concentration, heart rate at rest), three anthropometric parameters (body height, body weight, chest girth), and two motor tests (back strength, complex reaction speed). Seven out of eight diagnostic methods exhibit medium to high validity to discriminate between the different levels of performance development in the two sports groups. The investigated development of the performance characteristics is attributed partly to the inherited athletic disposition as well as to the different sport-specific training regimens of the two sports groups.

## Introduction

Worldwide, the predominant policies and structure of elite sport systems, and specifically in the youth departments, reflect the ‘talent account’ at least to a certain extent [1]. This covers, among other things, the search for talented players already at a young age who show natural abilities for a certain sport (discipline). Although the debate about validness of innate talent is lively and still ongoing, national sports associations are searching for the key-indicators for performance that can predict future successes. This early talent identification is considered crucial to keep up with the global medal race [2]. Nevertheless, even without over- or underestimating the role of innate talent, research focusing on the value of specific profiles of performance characteristics in elite youth athletes and the influence of training with respect to elite sports seems sensible. It will provide a better insight in the characteristics needed to perform at a high level and how efficient and effective trainings are to get there. Thus, it is paramount to diagnose the typical characteristics of the athletic profile and progress of the participants of different types of sports.

At this moment, it is clear that performance profiles are multi-dimensional and various characteristics should be taken into account [3–6]. Moreover, there is evidence that growth, development and specific training can influence a player’s profile especially at adolescence [7, 8]. Until now, most of the existing research concerning this topic is cross-sectional and focused on European and/or North-American athletes. These results might not be generalizable for adolescent athletes with a different origin. Players’ profiles and their response to training might be different across continents or even countries. Since Asia, and specifically China, can be considered as an ambitious competitor in sports, this study focused on the profiles of Chinese youth elite players of two different sports groups (i.e. swimming and racket sports) and their response to training. In long-term athletic development programs, training aims at the systematic optimization of performance prerequisites [9]. To allow for a close monitoring of the performance development of youth athletes in Shanghai elite sport school, the general sport administration and school officials introduced an assessment covering physiological, anthropometric, and motor tests during the whole time-course of school attendance of the talented youngsters. Although that these tests were suggested to monitor the development of performance characteristics over certain training stages, the development profiles can also help to discriminate players of different types of sports. In this study, these follow-up tests are used to investigate whether in the endurance sport swimming and the two speed-oriented racket sports table tennis and badminton, the performance development is also responsive to the intensive sport-specific training program. The insight in the discriminative ability and responsiveness to training of the different performance characteristics will support both concerns, talent identification and athletic development programs.

Elite endurance athletes as well as elite game sport participants are characterized by a markedly increased O_2_ transport capacity, hemoglobin concentration [Hb] and hemoglobin mass (Hbmass) [10, 11]. Hbmass and hemoglobin concentration [Hb] belong to the major limiting factors of maximum endurance performance. In various studies in endurance sports, a strong correlation between maximum oxygen uptake (VO2max) and Hbmass has been shown at all ages [12–14]. Furthermore, also in certain game sports, like e.g. field hockey, Hbmass is correlated with running endurance [15]. Therefore, swimmers, but also racket sports athletes aim to develop a high hemoglobin concentration [Hb]. However, even in primarily by aerobic endurance dominated sports, like e.g. cycling, no training effects were found after a 12-month training intervention in 11–15 yrs aged players [16] or after an 18 months`period of intensive endurance training in elite athletes aged 15–17 yrs [17]. These results lead to the assumption that erythropoietic adaptation might occur at a very young age or during late adolescence or that Hbmass is genetically determined. In contrast, some authors found a small (3%) increase in elite athletes during intensive training [16] or between training and recovery periods [18]. Thus, the question arises whether the high hemoglobin concentration [Hb]/high Hbmass observed in elite endurance athletes is primarily inherited and/or possibly achievable through long-term endurance training already during childhood and adolescence.

One marker of the physical fitness, that is sensitive to training during adolescence, is the heart rate at rest [19]. Thus, it is of major interest whether already at the preadolescent age there were differences between elite youth participants from the endurance-oriented sport swimming, and the more speed-oriented racket sports table tennis and badminton.

Additionally, the vital capacity (VC) is normally increased in endurance athletes, especially in swimmers [20]. Doherty and Dimitriou [21] found an 11% higher functional VC in male swimmers than in land-based athletes at the mean age of 15 years (M = 4,010 ml; SD = 1,200). As aerobic endurance does not play a dominant role in the individual game sports, table tennis and badminton, it is not surprising that even young, preadolescent swimmers exhibit a VC that comes close to that of adult table tennis (M = 4,420 ml, SD = 790) and badminton players (M = 4,000 ml, SD = 630) [22]. The superiority of swimmers over athletes from the individual game sports in VC was confirmed by Bloomfield et al. [23] as they found better values for 11–12 years old swimmers in comparison to tennis players of the same age. Also Zhao et al. [24] found VC values of M = 5,071 ml (SD = 863) in Chinese U15/U16 age group swimmers, which were significantly higher than in a group, comprising youth athletes from five different other sports. In contrast, in that study VC was systematically lower in table tennis athletes (M = 3,823 ml; SD = 423). In combination with the VC, the chest girth (CG) is an adaptation effect of the respiratory muscles in well-trained swimmers [25]. For a Chinese U15/U16 age group of swimmers, Zhao et al. [24] reported a chest circumference of M = 90.3 (SD = 4.3), which was significantly greater than in the group of the youth athletes from the other five sports.

In racket sports, speed parameters, like the reaction time (RT), are suggested to play an important role [26]. As a general result, one can confirm the assumption that elite athletes in most sport disciplines similar to racket sports differ from non-athletes when performing generic, single, and elementary reaction tasks (for table tennis, see [27]). Nevertheless, there is but little evidence for the validity of a simple eye-hand RT assessment to distinguish between elites from different sports [28].

Finally, in jumping disciplines like basketball [29] or volleyball [30] maximal dynamic back strength (BS) turned out to be a relevant predictor of sport performance. This is explained by that the deadlift exercise contributes to the activation of the m. semitendinosus during leg extension and the push-off movement [31]. In swimming [32], the power of the squat movement, which is highly dependent on dynamic BS, is a relevant predictor for the lunge speed and swimming power, respectively. Zhao et al. [24] reported on a higher level of dynamic BS in U15/U16 Chinese table tennis athletes compared to a group of other sports. Furthermore, the high reliability of the deadlift test (ICC = 0.99; [33]) allowed for the use of this measurement in all three sports groups included in this study.

On the basis of such findings, the purpose of this study was to investigate the profile and its response to training of adolescent Chinese elite athletes of swimming and racket sports. It is hypothesized that these youth players already show a sport specific development profile of certain physiological, anthropometric and/or motor performance characteristics which is in line with the specific requirements of each of the particular sports. Moreover, it is hypothesized that the response to the different sport-specific training regimens will also lead to increasing differences in the development of the athletic make-up.

## Materials and methods

### Participants

The Shanghai Elite Sport School focuses on five different sports branches, of which the investigated two sports groups are the largest at the respective age. Whereas the investigated endurance sports group consists of the open water and bassin swimmers, the group of speed-oriented sports embraces the comparatively homogeneous racket sports group of the investigated table tennis and badminton players. Besides these two groups, the Elite Sport School also promotes combat sports which includes the more heterogeneous judo and fencing athletes, as well as the "big" sports games which consist of baseball, basketball, and volleyball players, and a selection of somewhat older sprint running, hurdling, high and long jump, pole vault and decathlon athletes.

In total, 39 youth male Chinese elite athletes aged between 12–14 years started to take part in the 2-years follow-up assessment. Due to injuries or illness 18 athletes could not take part in all five waves of the repeated measurements, so that n = 21 participants completed the full study (swimming = 10, and racket sports = 11 that is 7 table tennis and 4 badminton players; mean age 12.14 ± 0.62 years, age range 11–13 years) participated in this study. All participants were part of the Shanghai Elite Sport School, and regularly participated in the sports investigated in one 3-hours training session from Monday to Friday and two 3-hours training sessions on Saturday which amount to 21 hours total training time per week, as well as in frequent competitions. The mean training time per week of the youth athletes was M = 20.8 hrs/w (not including school sports) over the entire study period. According to the coaches`training log the swimmers weekly training volume of 60–75 km is apportioned to six days per week and equivalent to about 15 hrs/wk of swimming. Over the course of the training year the swimming program consisted of 67 percent of aerobic endurance training, 28 percent of mixed aerobic-anaerobic, and 5 percent of anaerobic-lactic training. In addition to the swimming sessions, the athletes conducted 2–3 of strength and conditioning sessions per week (4–6 hrs/wk). Although the training load of the in-water and dryland training could be differentiated according to more precise categories of training intensity, at least the total training volume in both forms of training is in line with the data reported by Pollock et al. [34] for British national caliber swimmers. Furthermore, the swimmers weekly training volume complies with the time standard of 21 training hours of the racket sports players. The highly comparable total amount of training load and a training quality on international level of the swimmers and racket sports groups is secured by the official timetable of the Elite sport school training and education program, and the professional expert staff, respectively. Like in the swimmers, the racket sports groups`training program included 15 hrs per week of 67 percent sport-specific technical-tactical training and 33 percent match play activities, accompanied by 2–3 strength and conditioning sessions amounting to 4–6 hrs/wk. In both sports groups the high volume of sport-specific training should enhance the development of the aerobic endurance which in swimming is needed for sustaining swimming speed [35], and in table tennis and badminton for quick recovery (see [36, 37], as well as [38–40], resp). The strength and conditioning exercises in both sports groups take place at the same high standard level and is focused predominantly on core strength and the explosive power of arms and legs. So, in both sports groups the young elite athletes should demonstrate considerable training adaptations in the compared physiological performance characteristics, body dimensions, and motor abilities [41, 42].

The mean training experience at the beginning of the study was two years. All athletes were performing at a high level in their respective sport, representing China and/or the Shanghai province in international competitions.

As non-athletes are not part of the Shanghai Elite Sport School, a control group was not at hand. To allow for the conclusion that the investigated development of the 12–14 years old youth athletes could not be related alternatively to the natural grow of Chinese teenagers, we could refer our initial assessment parameters at least to actual data of representative Chinese surveys on body height and weight [43], vital capacity [44], and hemoglobin concentration [45].

The participants were recruited according to the ethical standards of the Shanghai University of Sports (SUS). This study was carried out in accordance with the recommendations of “Science Research Ethics Committee at the Shanghai University of Sport” with written informed consent from all subjects. All subjects gave written informed consent in accordance with the Declaration of Helsinki. The protocol was approved by the “Science Research Ethics Committee at the Shanghai University of Sport.” All athletes’ parents were informed about the protocol of this study, which was outlined in an information letter. No data collection took place without parents’ consent.

### Design of the study

In our study, a mixed cross-sectional and longitudinal design was used to allow for the detection of differences in the athletic development pathways exhibited by the two different sports groups, and to inform about selection and/or training effects. Both sports groups were monitored for two years by means of 5 test waves, with 2 visits (March and September) per year. All 21 athletes completed the study and performed all 8 tests.

### Procedures and protocols

At each visit, all athletes completed a questionnaire that asked about their training volume and frequency, injuries, and illnesses within the previous three months. None of the participants taking part in all five test waves showed any severe injuries or illnesses that would have been a reason for exclusion. For both groups, the same parameters were measured during one visit by expert sport school staff members; body height (BH) and body weight (BW), resting heart rate (HR), CG, Hb concentration [Hb] and VC, maximum dynamic BS and eye-hand RT. All tests were conducted on the same day in both the gym and sports science laboratory on campus. The testing started at 10 a.m., and all athletes refrained from strenuous exercise one day prior to the test session.

#### Physiological characteristics

VC (in ml; high precision digital electronic spirometer, Donghuateng Sports Apparatus Ltd, Beijing, CN) and hemoglobin concentration [Hb] (in mg per l; HemoCue Hb 201; HemoCue AB, Angelholm, Sweden) were diagnosed by medical personnel of the Shanghai University of Sport. The typical error for the expiratory VC measurement is 1.7% [46]. Arterialized blood samples were taken from a hyperemized earlobe to determine the [Hb]. [Hb] was assessed photometrically (HemoCue® HB201+, HemoCue AB, Sweden). The typical error for [Hb] measurement with this device is as accurate as CV < 1.0% (limits of agreement –1.28 to 0.20 gr/dL; [47]). The HR at rest was recorded in all five periodical measurements for 2 min immediately after awakening in the morning by positioning an H7 Bluetooth HR strap (Polar Electro, Kempele, Finland) on the chest.

#### Anthropometrics

BH were measured to the nearest 0.1 cm with a mechanical height tester (Donghuateng Sports Apparatus Ltd, Beijing, CN), BW to the nearest 0.1 kg (calibrated Seca Alpha 770), and CG to the nearest 0.1 cm with a circumference ruler (Donghuateng Sports Apparatus Ltd, Beijing, CN) were measured according to standardized test prescriptions that ensure a high reproducibility [48, 49].

#### Motor performance prerequisites

Maximum dynamic BS (in kg; measured by a power deadlift) and simple RT (in ms; PsyTech Sports; Xinyi Electronic Technology Company, Shanghai, CN) were tested by expert staff members from Shanghai elite sport school. Before the dynamic BS test, subjects performed a warm-up that consisted of cycling and dynamic stretching, followed by the standardized one repetition maximum (1 RM) deadlift [50]. During the test, the standardized procedures for the one repetition maximum (1 RM) deadlift was followed [50]. A low-intensity set of 5–10 repetitions was performed using 40–60% of the perceived 1 RM. After a 1-min rest, subjects performed a set of 2–3 repetitions at 60–80% of the perceived 1 RM. Subsequently, subjects performed 3–5 maximal trials, followed by an assessment of 1 RM deadlift strength. For the power deadlift, Dorrell et al. [51] reported typical errors varying for all variables between two visits from low to moderate (range 0.6–8.8%).

In the simple RT assessment, the test device was prepared to measure the time of a simple finger movement response to light stimulation. The participant sat in front of the test instrument, placed his right index finger on the button, and pressed the button when the red light was on. The measurement included 20 repetitions, and the average value was calculated and used for all further data analysis.

### Data analyses

All data were analyzed with SPSS (Version 25.0; SPSS Inc., Chicago, IL, USA). To compare the data from the two sports groups, the group means of each parameter were calculated for each of the five test waves. Subsequently, these data were used in a General Linear Model (GLM) with a 2*5 repeated measurements ANOVA to determine the differences between the five subsequent test waves (factor time), and possible differences in the development of the 8 parameters between the two groups (factor sports), as well as interactions between groups and development. In all analysis the significance level was set at *p* < 0.05. According to the rules of thumb proposed by Miles and Shevlin [52], effect sizes (partial η^2^) were interpreted as being small (< .06), medium (< .14) and large (> .14). Although there were no systematic statistical differences among the two sports groups in regard to calendar age, in the GLM analysis the age of the participants at the beginning of the study (test wave 1) was used as a covariate to exclude even slight influences of this factor on the parameters investigated.

## Results

Descriptive statistics for all 8 variables of the 5 test waves used in the GLM can be found in Table 1.

**Table 1:** Physiological, anthropometric, and motor performance data.

	Initial test (9/2016)	Test wave 2 (3/2017)	Test wave 3 (9/2017)	Test wave 4 (3/2018)	Test wave 5 (9/2018)
**Swimming group (n = 10)**					
Age (months)	145.8 ± 7.2	151.8 ± 7.2	157.8 ± 7.2	163.8 ± 7.2	169.8 ± 7.2
Hemoglobin concentration (gr/l)	128.5 ± 11.1	129.8 ± 12.5 #	132.3 ± 8.3	* 137.2 ± 8.7 ###	* 138.1 ± 11.0
Vital capacity (ml)	3796 ± 645 ###	** 4200 ± 857 ##	** 4486 ± 1064 ##	*** 4823 ± 1081 ##	*** 4911 ± 11.30 ##
Heart rate at rest (bpm)	61.9 ± 7.0	63.8 ± 7.5	61.7 ± 7.3 ##	62.6 ± 4.7 ###	61.7 ± 5.9 ##
Body height (cm)	165.1 ± 9.4 ##	*** 168.5 ± 10.6 ##	*** 171.9 ± 10.7 #	*** 175.4 ± 10.2 #	*** 177.9 ± 9.5 #
Body weight (kg)	57.2 ± 14.3 ##	57.9 ± 13.2 ##	58.9 ± 14.6 #	62.4 ± 15.0 #	63.7 ± 14.2 #
Chest girth (cm)	81.0 ± 7.5 ##	** 83.7 ± 7.3 ##	** 83.9 ± 8.0 #	*** 86.4 ± 8.9 ##	*** 87.5 ± 8.2 ##
Dynamic back strength (kg)	68.6 ± 10.1	* 74.7 ± 15.3	** 79.6 ± 17.3	** 89.8 ± 21.7	** 90.3 ± 19.8
Eye-hand reaction time (ms)	210 ± 27	199 ± 18	197 ± 19 #	222 ± 26	200 ± 23
**Racket sports group (n = 11)**					
Age (months)	145.6 ± 8.0	151.6 ± 8.0	157.6 ± 8.0	163.6 ± 8.0	169.6 ± 8.0
Hemoglobin concentration (gr/l)	119.7 ± 12.7	117.1 ± 9.7 #	122.3 ± 17.1	119.6 ± 10.4 ###	** 132.8 ± 14.5
Vital capacity (ml)	2686 ± 335 ###	** 2946 ± 399 ##	*** 3059 ± 478 ##	*** 3345 ± 414 ##	*** 3432 ± 477 ##
Heart rate at rest (bpm)	70.5 ± 10.1	68.1 ± 5.7	71.2 ± 6.4 ##	70.4 ± 3.8 ###	69.4 ± 4.0 ##
Body height (cm)	154.9 ± 8.7 ##	*** 157.7 ± 9.1 ##	*** 161.4 ± 9.6 #	*** 165.1 ± 9.4 #	*** 169.0 ± 8.9 #
Body weight (kg)	42.4 ± 9.1 ##	** 46.3 ± 9.2 ##	** 47.5 ± 9.7 #	*** 51.1 ± 9.0 #	*** 53.2 ± 9.2 #
Chest girth (cm)	71.7 ± 7.6 ##	** 73.9 ± 8.0 ##	*** 76.0 ± 7.1 #	*** 78.1 ± 6.6 ##	*** 79.8 ± 6.7 ##
Dynamic back strength (kg)	57.1 ± 17.0	** 67.9 ± 19.3	*** 71.1 ± 14.0	*** 77.2 ± 18.6	*** 85.2 ± 17.3
Eye-hand reaction time (ms)	223 ± 34	213 ± 32	219 ± 21 #	229 ± 17	223 ± 32

Values are means and standard deviations (M ± SD). Significance of differences within the two sports groups between initial and following values

*p < 0.05

**p < 0.01

***p < 0.001.

Significance of differences between the swimming and racket sports groups

^#^p < 0.05

^##^p < 0.01

^###^p < 0.01.

### Physiological characteristics

**Fig 1:**
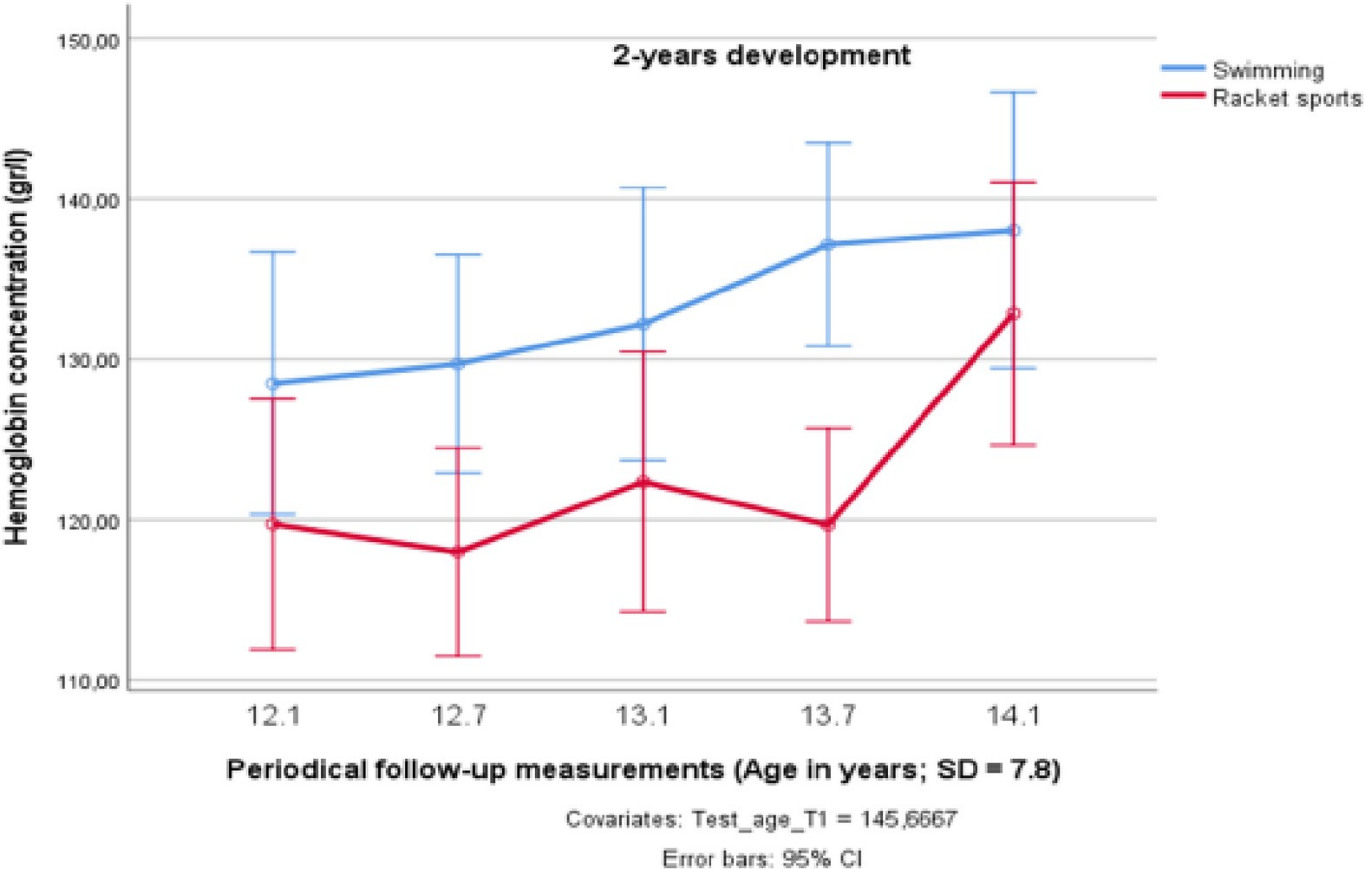
Changes in Hb concentration over two years in preadolescent male elite athletes from swimming and racket sports.

The results of the General Linear Model (GLM) with age as a covariate for [Hb] in the athletes from both sports groups are presented in Fig 1. When the two sports groups were compared, [Hb] developed in a slightly different manner, and appears to have increased in a more linear pathway in the swimmers, whereas the racket sports participants exhibit a time-lagged sharp increase in the development of [Hb] over time, which took place on a significantly lower level (F_1;18_ = 7.983; *p* < 0.05 partial η^2^ = 0.307). Nevertheless, no significant interaction effect between the time course of [Hb] development and the sport performed could be detected (F_4;15_ = 1.383; *p* = 0.249; partial η^2^ = 0.071).

**Fig 2:**
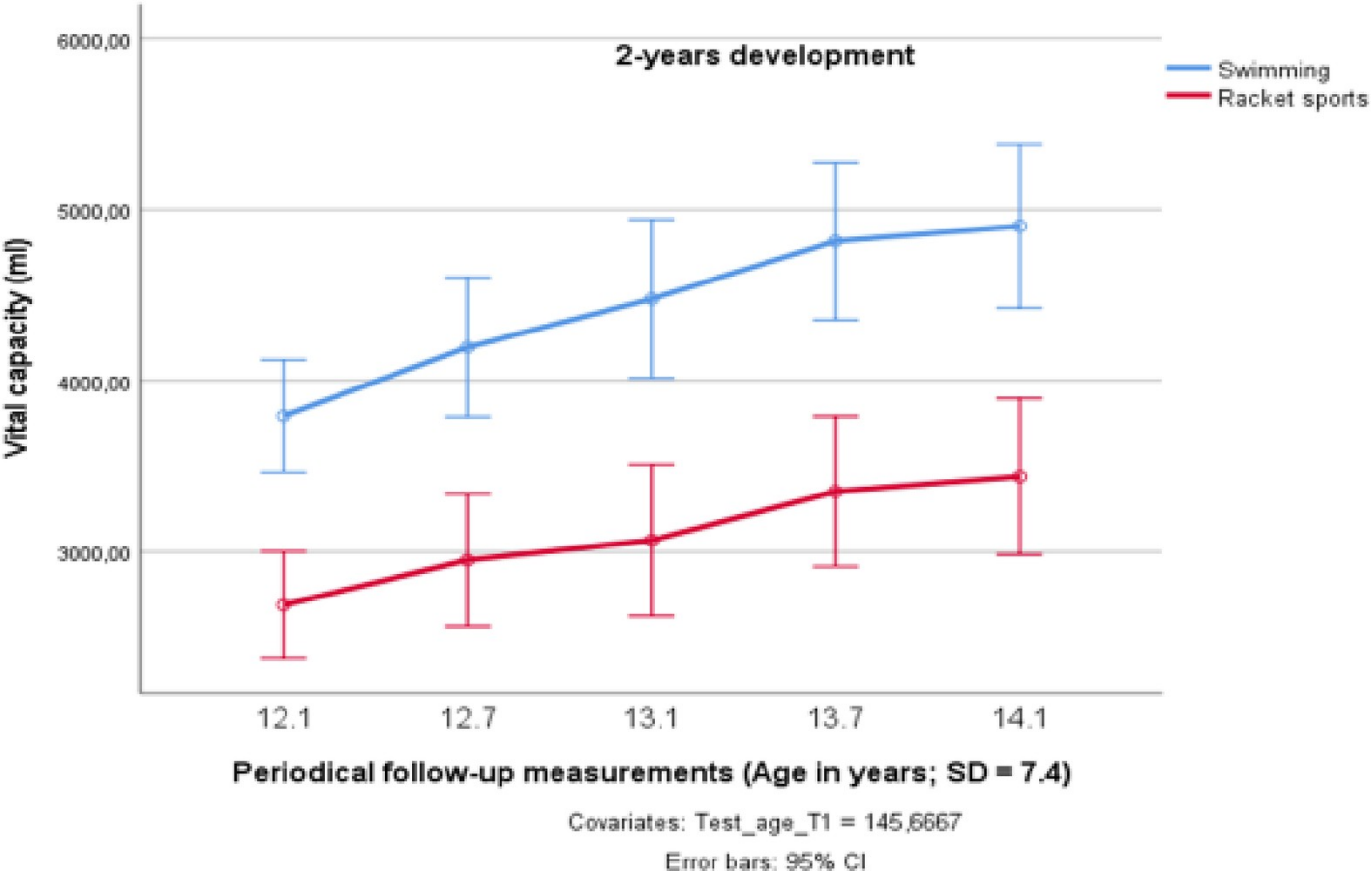
Changes in vital capacity over two years in preadolescent male elite athletes from swimming and racket sports.

A greater development of the VC took place in the group of swimmers, which was found to be significantly different from that of the racket sports groups (F_1;18_ = 24.521; *p* < 0.001; partial η^2^ = 0.577), which also showed an increase, but again on a lower level (Fig 2). Overall, the investigated ten swimmers improved their VC by 31.5%. This large effect led to a maximum VC at the end of the study period of almost five liters (M = 4,911 ml). Although the increase in the racket sports athletes’ group by 32.5% was almost the same, the endurance athletes`maximum VC was more than 1.5 liters higher (Table 1). As the development in the swimmers`group took place on a higher level, a significant time by group effect (F_4;15_ = 2.665; *p* = 0.039; partial η^2^ = 0.129) could be detected.

**Fig 3:**
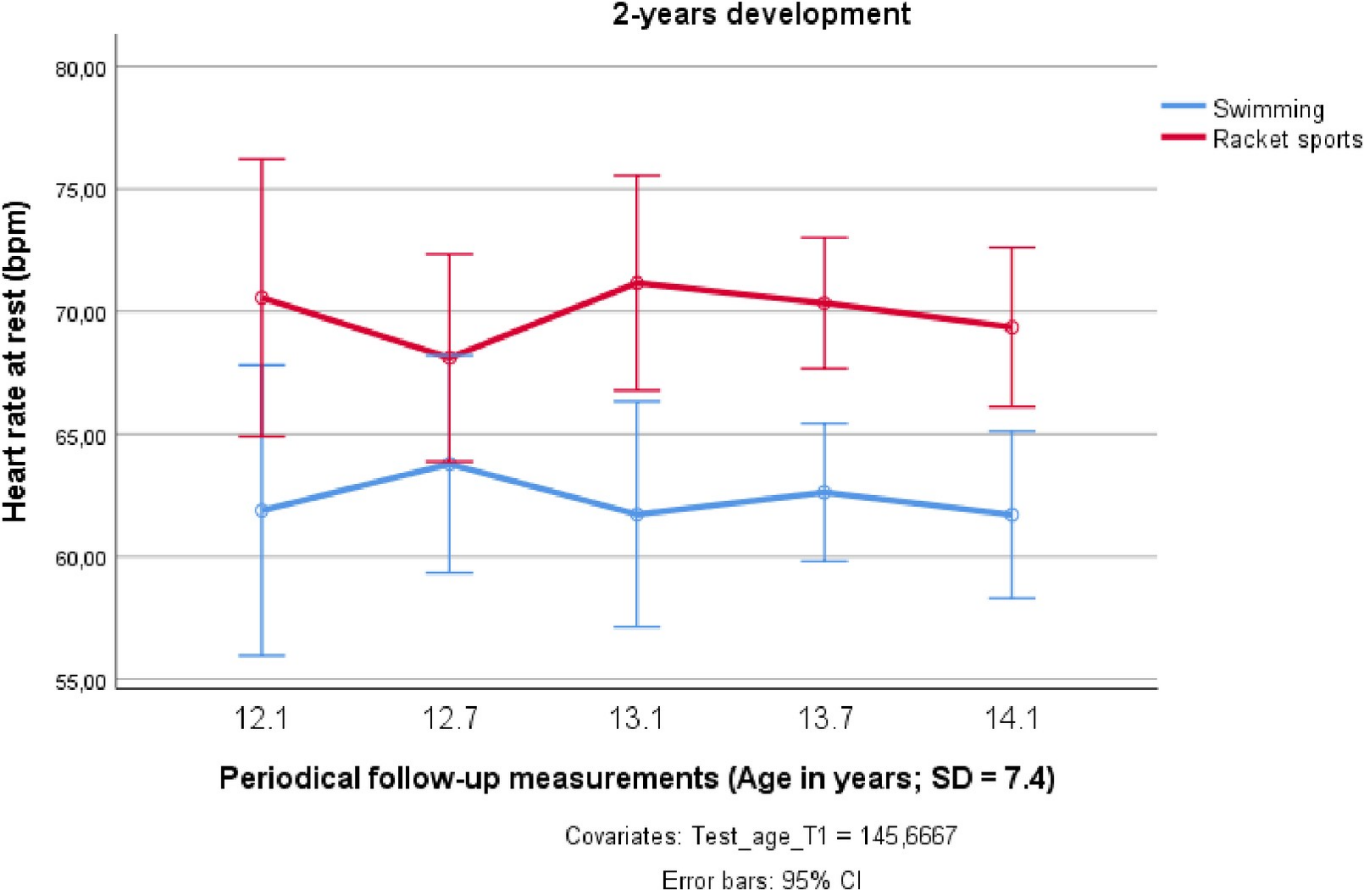
Changes in heart rate at rest over two years in preadolescent male elite athletes from swimming and racket sports.

The resting HR in both sports groups did not decrease over the investigated 2-years preadolescent time span, and remained more or less on the same level (Fig 3). The significantly lower HR at rest of the Chinese swimmers persisted over the whole investigation period (F_1;18_ = 13.674; *p* < 0.01; partial η^2^ = 0.432).

### Anthropometrics

Changes in the anthropometric parameters of the two groups during the study period are shown in Table 1. The increase of BH in the pubertal athletes of both sports groups by approximately 6–7 cm per year did not reach significance (F_4;15_ = 2.348; *p* = 0.062; partial η^2^ = 0.115), although the swimming group was significantly taller (F_1;18_ = 11.765; *p* < 0.01; partial η^2^ = 0.395). In the generally heavier swimmers (F_1;18_ = 8.212; *p* < 0.05; partial η^2^ = 0.313) body mass showed a small increase in the first year, followed by a higher increase in both groups of more than 5 kg during the second year (Table 1).

**Fig 4:**
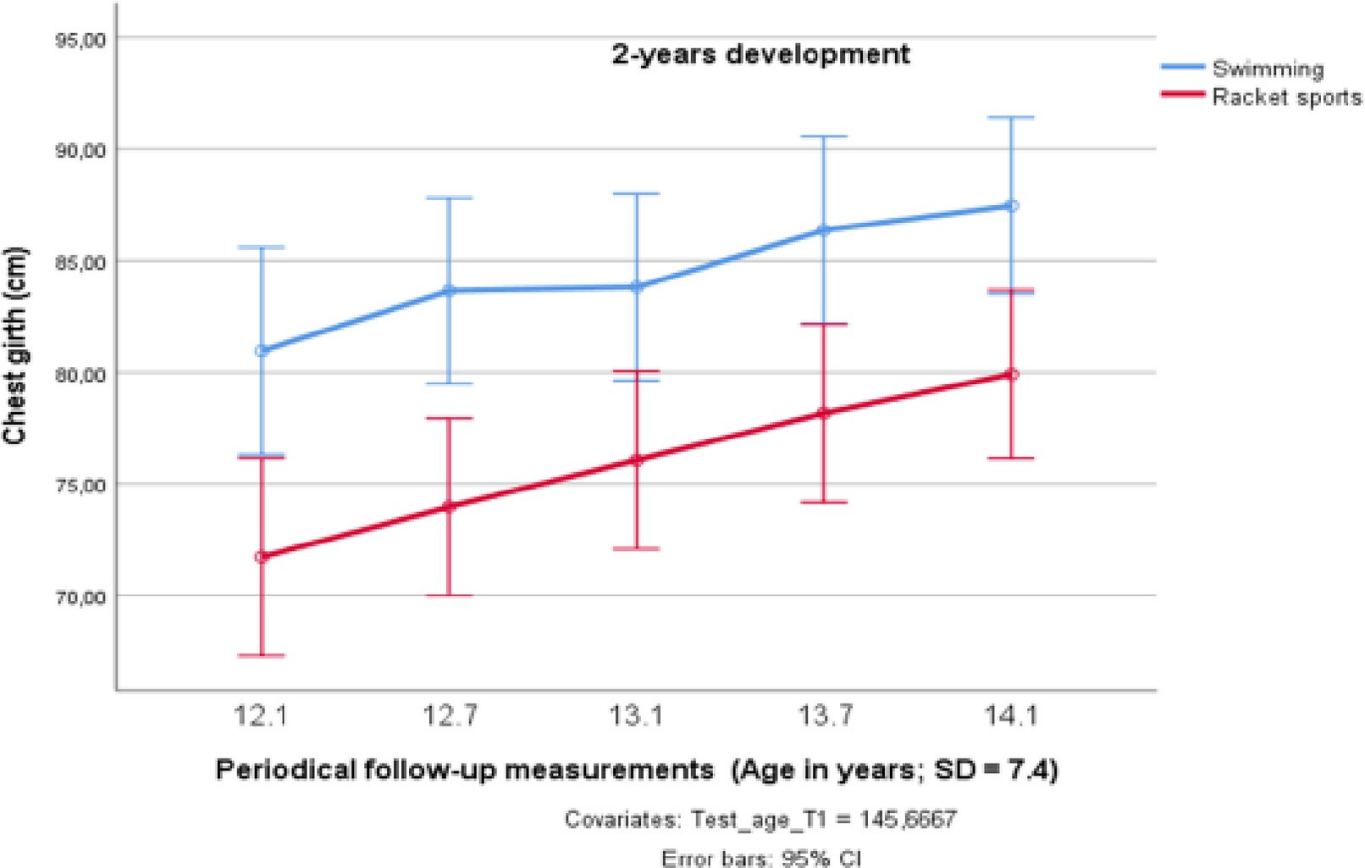
Changes in chest girth over two years in preadolescent male elite athletes from swimming and racket sports.

CG increased by 3–4 cm in the first, and also in the second year (see Table 1), thus the overall increase in the total group was significant (F_4;15_ = 2.989; *p* < 0.05; partial η^2^ = 0.142). As Fig 4 shows, the CG of the ten swimmers was already greater in the beginning of the monitoring and their lead remained stable over the whole two-year follow-up period. Although the swimmers presented a much larger upper body than their racket sports counterparts (F_1;18_ = 9.790; *p* < 0.05; partial η^2^ = 0.352), the shape of the course of the developmental pathway did not vary significantly between the two sports groups.

### Motor performance prerequisites

**Fig 5:**
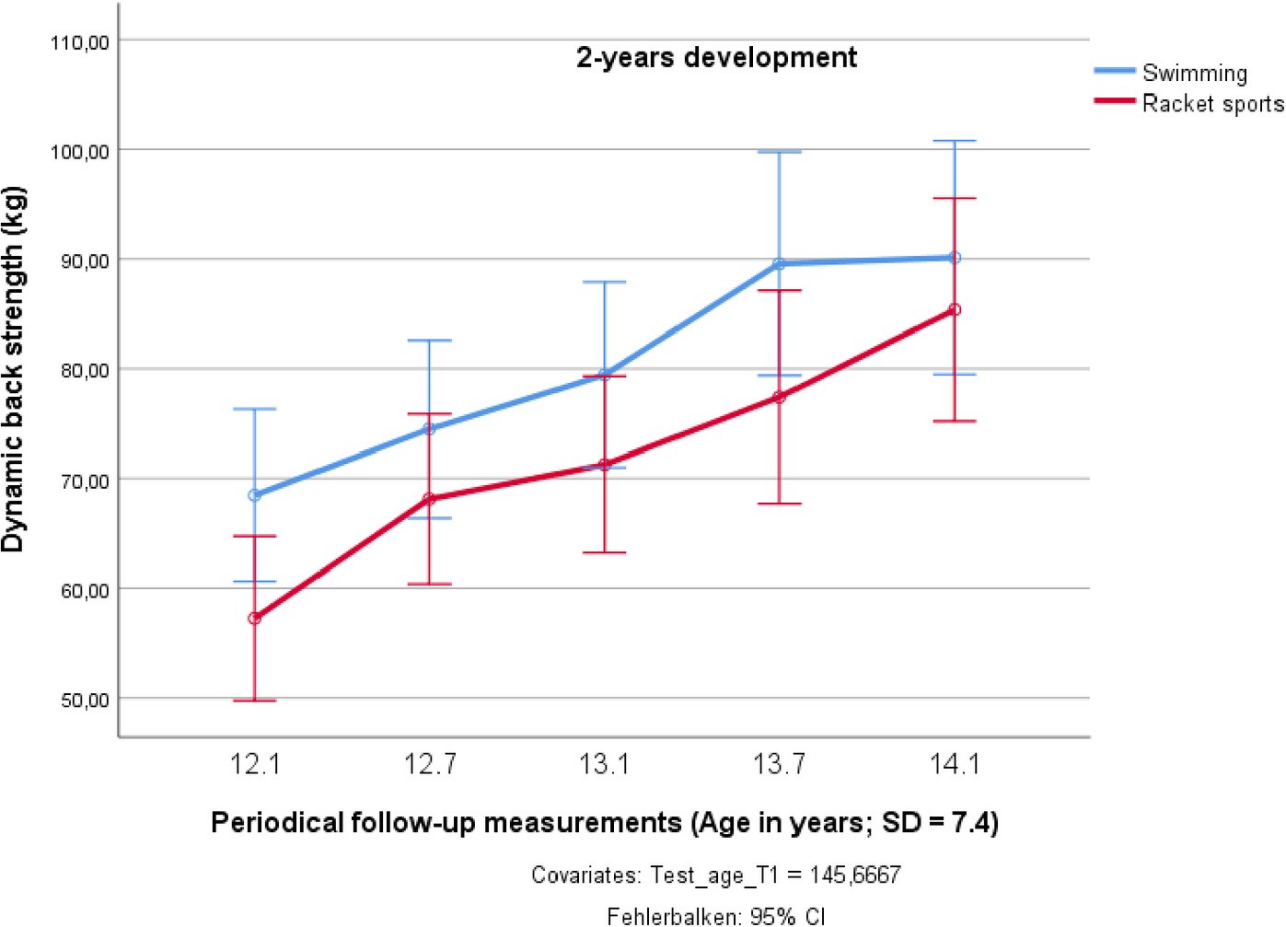
Changes in dynamic back strength over two years in preadolescent male elite athletes from swimming and racket sports.

The swimmers`group performed the BS test constantly on a higher level, but this difference was not significant (F_1;18_ = 2.844; *p* = 0.109; partial η^2^ = 0.139). Also, the development of the maximum dynamic BS was not systematically different between the racket sports and swimming groups, although there seemed to exist a certain levelling of the BS development during the last half year in the swimming group. In the racket sports group, the dynamic BS increased over the two years by 44.0%. Although the ten swimmers exhibited the highest maximum BS over the whole investigation period, their relative gain in BS was less (34.2%; see Fig 5).

**Fig 6:**
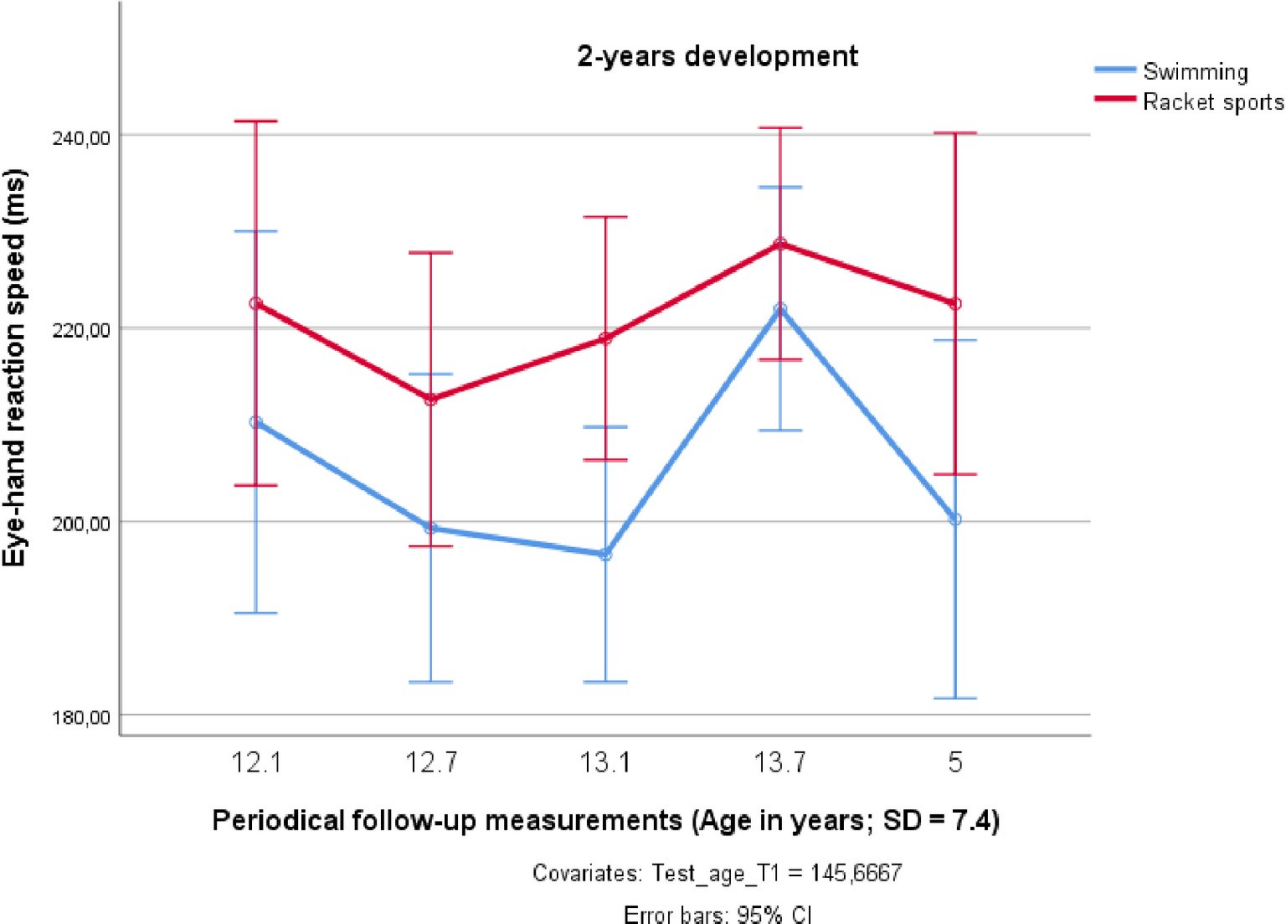
Changes in eye-hand reaction time over two years in preadolescent male elite athletes from swimming and racket sports.

The development of the single RT exhibited a rather unsystematic pattern (see Fig 6). It came as a surprise that the swimmers performed significantly better in the eye-hand RT test (F_1;18_ = 7.718; *p* < 0.05; partial η^2^ = 0.300), even though these athletes did not improve much over time (Table 1). As Fig 6 demonstrates, there was no significant interaction between the RT development and the sports performed (F_4;15_ = 0.444; *p* = 0.776; partial η^2^ = 0.024).

## Discussion

The aim of the study was to monitor the development of anthropometric, physiological and motor performance prerequisites in elite youth athletes for a period of two years to investigate the influence of systematic and sport-specific training on these variables. Based on the results of this current study the development pathways of the anthropometric, physiological, and motor performance prerequisites assessed in the elite sport school in Shanghai, China, are not systematically different from Caucasian athletes investigated in comparable studies in the literature.

In regard to body height and body weight, the swimmers`group was at the age of 12 years about 11 cm taller and 11 kg heavier than untrained male children of the same age, when compared to the representative data of Zong et al. [43] from the large coastal cities in China. On the other hand, the racket sports players did not exhibit a difference in body height, but were about 4 kg lighter than non-athletes of the same age. Body height in swimming is widely recognized as an important talent factor and performance characteristic [53]. In table tennis, a low body weight as well as a short lower leg length [24] might enhance primarily the players`agility which plays an important role in elite table tennis performance [54].

The mean values for the [Hb] of the athletes of the three sports groups showed an average yearly increase of 4.4% in the swimmers, and 6.5% in the racket sports players, which was similar to published data for Hbmass, showing a mean value of ~430 g in 11–13-yr-old boys and ~370 g in 12–13-yr-old girls and boys [12]. Despite the general increase in hemoglobin mass with age, Prommer et al. [11] did not find an independent statistical effect of age on Hbmass because, in their study, the effects fully overlapped with those of lean body mass (LBM) in such way that a 1 kg increase in LBM was associated with a 14.7 g increase in Hbmass. As literature has stressed the close relationship between the circulating testosterone levels and [Hb] [55], this leads to the hypothesis, that also the increase of [Hb] in young athletes is primarily affected by androgens [56–58]. This supports the assumption that the sharp increases in [Hb] of 15.8 percent in the racket sports group between the age of 13.7 and 14.1 years observed in our study seem to reflect puberty-associated changes in erythropoiesis and were directly linked to an increase in testosterone levels in the male athletes. In contrast, it remains unclear why such a nonlinear development of [Hb] was not found in the swimmers of the endurance group who showed an almost linear increase over the two-years period.

The impact of the endurance training, itself, on accelerated erythropoiesis has been discussed and the findings are controversial. According to Steiner and Wehrlin [59], who compared the Hbmass values of elite endurance athletes at various adolescent ages and concluded that training at an age younger than 16 years appeared to have had negligible effects with respect to enhanced erythropoiesis, our findings support the hypothesis that, during the investigated 2-years training period during adolescence, genetic predispositions more than the different sports-specific training regimens had an essential impact on [Hb]. Our results are also in line with Ulrich, Bartsch, and Friedmann-Bette [17], who monitored the Hbmass of 15–17-yr-old boys and girls during a 1.5-yr training period, and found a 15% higher Hbmass in the trained subjects than in the untrained subjects, but no systematic training effects. Very similar results were demonstrated by Eastwood et al. [16] for 11–15-yr-old boys and girls after a 1-year training period, showing 10% higher values in the athletic group (cyclists) compared to non-athletes, which the authors attributed to the normal maturation process and not to the training, itself. These results coincide well with the increase of 10–13 gr/l in [Hb] observed in this study for the 12.1–14.1 years old age group (Table 1), corresponding to a change of 7.5% in the swimmers`, and 10.9% in the racket sports group over the 2-years training period. This training effect exceeds the development of 5.1% of [Hb] in untrained Chinese children between 12–14 years of age, that was reported by Song et al. [45] in his national overview.

However, when comparing the various training regimens during the training period, we cannot determine whether this increase represents a sports-specific training effect, or whether the higher values of the young swimmers are due to a selection process that favors children with a naturally high [Hb] in this endurance sport [16, 17]. As a similar increase of [Hb] occurred in the racket sports group during the pubertal phase investigated here, we hypothesize a high basic genetic impact and a genetically determined influence of endurance training on [Hb], as the gains per year have not been higher in the swimming group. Alternatively, it can be speculated that a higher effect of the swimmers endurance training regimen on [Hb] had been masked by a parallel increase of blood plasma volume due to erythropoiesis [60].

In the HR at rest, similar results have been observed in former studies on preadolescent elite athletes in sports that require particularly extended cardiac demands in response to an overall endurance-oriented training program. So, it is interesting to note that in the investigated Chinese age group swimmers, the average resting HR was about 7 bpm lower than in the national calibre athletes of the same age (*M* = 14.3 yrs; *SD* = 1.0) from a Lithuanian elite sports school, where Kamandulis et al. [61] diagnosed M = 68.6 ± 6.9 bpm. As the resting HR is one marker of the functional status of the organism, it can be assumed that the lower values of the Chinese youth athletes resulted from a more demanding training regimen that exceeded the weekly training volume of the European swimmers by 2–3 training sessions that is about 5–6 hours per week.

A very similar picture as that for [Hb] and resting HR development was found for VC with respect to the developmental change during the age period of 12.1 to 14.1 years investigated in this study. As expected, the greatest development of the VC took place in the endurance group. Here, the investigated ten swimmers improved their VC by 31.5%, which led to a maximum VC at the end of the study period of almost five liters (M = 4,911 ml). Although, the increase in the racket sports athletes by 32.5% was almost the same, the endurance athletes`maximum VC was about 1.5 liters higher (Table 1). The greater development found in the swimmers agrees with the previous studies of Lazovic et al. [25], who reported higher lung volumes in endurance sports compared to skill, mixed, and power sports. Furthermore, Mercier et al. [62] stressed that, in 10–14 years old swimmers, the increase of V02_max_ is strongly linked to training volume. As the training volume of the circumpubertal swimmers investigated in our study is higher than the amount of 14 h per week reported for the group with the highest gain in V02_max_ by Mercier et al. [62], it can be assumed that the superior values of the Chinese swimmers compared to their racket sports counterparts resulted, primarily, from their sports-specific training regimen. The main influence factor that might be responsible for the higher gains in VC in the swimmers compared to the group of the racket sports participants, exhibiting a quite similar development of their VC on a lower level, can be seen in the strengthening effect of the underwater pressure on the respiratory muscles of the swimmers [63]. So, it is not a surprise that the VC of the swimmers`group already at the beginning of our investigation period exceeded the mean value of 12.5 years old untrained subjects from the city of Shanghai (M = 2517 ml; [44]) by 50.8%., while in the racket sports group this difference was 6.7%.

The strengthening effect of water pressure on the respiratory muscles of swimmers not only enhances swimming performance but might also contribute to the greater CG in the swimmers of the endurance group. This mechanism is not only typical for swimmers but can also be detected in fin swimming [64] and scuba diving [65]. The CG of the ten swimmers of the endurance group was already greater in the beginning of the study and remained stable over the whole 2-years follow-up period.

The relevance of dynamic BS was reported in a variety of different sports. In game sports like basketball [29] or volleyball [30], maximal dynamic BS turned out to be a relevant predictor of sport performance, and also reduced injury prevalence [66]. In swimming, Morouco et al. [32] found that the power of the squat movement is a relevant predictor not only for lunge speed and swimming power, but also enhances swimming endurance via a more stable prone position which reduces drag resistance considerably for all swimming velocities. Besides its validity, the high reliability of the deadlift test (ICC = 0.99; [33]) allowed for the use of this measurement in both sports groups of this study.

The finding of this study, which concludes shorter RT in a single eye-hand coordination task for the group of the swimmers needs further investigation as it cannot be confirmed by the existing literature.

### Study limitations

Our study has several limitations. The first limitation is the relatively small sample size, which was a consequence of the inclusion of only elite athletes. This implies that members of the investigated age group of 12-14-year-old male athletes cannot be numerous. Besides that, the small number of athletes from the two sports groups, resulted also from the short-term drop-out rate of 18 participants shortly before any one of the five single measuring dates due to injuries, illness or other causes.

The second limitation is the study’s narrow focus on only male youth athletes. This was due to feasibility reasons. At the moment of testing, the number of elite female athletes was even smaller compared to the males. This would have caused further statistical restrictions. oreover, we expected a different influence of the gender-specific athletic make-up on the sports-specific performances of male and female youth athletes in the two sports groups. For this reason, it seems invalid to group male and female athletes together.

Furthermore, the lack of a control group participating in a more general training program on a non-elite level, monitored over the same time period and with the same frequency as the elite youth athletes`groups, makes it difficult to distinguish between real training effects and genetic predispositions. Nevertheless, the data collected in this study provide valuable insights into the changes in [Hb], VC, and CG, as well as BS development, under the relatively intensive training regimen in an elite youth sport school in China. Although that the elite sport school system secures a comparable maximum of total training load and a training quality on the international standard level in the players from different sports, in future studies a deeper insight into the details of the sports-specific training load set-up and the progression and periodization of the training volume and intensity over this important age period in the long-term athletic development is desirable. Besides more precise information on the kilometers swum in different intensity categories in the swimmers group, and on the exercise duration in specific training content categories, like e.g. technical and tactical training drills and match play in the racket sports group, further information on the individual training response is warranted. So, besides measures of resting, exercise, and recovery heart rate during the training sessions [67], also daily training logs, psychometric questionnaires [68], and frequent performance testing of endurance, speed and strength parameters [69] may offer a more complete solution to diagnose the long-term performance development in athletes participating in aerobic- and strength-oriented sports.

## Conclusion

In general, elite youth athletes of the different sports groups (swimming and racket sports) from China between 12–14 years of age exhibit similar development profiles of physiological, anthropometric, and motor performance prerequisites profiles, compared to North American or European athletes. [Hb] and VC of Chinese male youth athletes linearly increase between the ages of 12 and 14 years, showing a mean increase of 5% per year, not only reflecting their sports-specific response to training, but also the impact of testosterone production during the onset of puberty. These age-related changes in [Hb] are mainly promoted by the development of body mass, although long-term training exerts additional effects. However, this study provides evidence that the higher [Hb] found in the endurance-trained swimmers between the age of 12 and 14 years is not primarily due to the sports-specific training regimen alone, but also to genetic preselection. From our study, there is no evidence that the talent recruitment strategies or the sports-specific training regimens administered in a typical elite sport school in China cause different development pathways in Chinese youth athletes when compared to North-American or European players, and thus need to be changed. Nevertheless, the heterogeneity of the intra-group samples in the two racket sports disciplines table tennis and badminton summed-up as similar sports types, require a cautious interpretation of our results. Also, the focus on solely male athletes point to a need for further investigations in the talent development process-up of elite youth sport cadres. Furthermore, a greater variety of motor tests including speed, endurance and flexibility tests in the youth athletes`assessments were reasonable.

## Supporting information

S1 Data (XLSX)

## References

[1] Baker J. & Wattie N. (2018). Innate talent in sport: Separating myth from reality. *Current Issues in Sport Science (CISS)*.

[2] Faber I.R. (2019). The talent quest–comment on Baker. Accepted for publication in: *Current Issues in Sport Science (CISS)*. 10.15203/CISS_2019.103

[3] Elferink-Gemser M.T., Visscher C., Lemmink K.A.P.M. & Mulder T. (2007). Multidimensional performance characteristics and standard of performance in talented youth field hockey players: A longitudinal study. *J Sports Sci*., 25(4):481–489. 10.1080/02640410600719945 17365535

[4] Opstoel K., Pion J., Elferink-Gemser M., Hartman E., Willemse B., Philippaerts R., Visscher C., et al (2015). Anthropometric Characteristics, Physical Fitness and Motor Coordination of 9 to 11 Year Old Children Participating in a Wide Range of Sports. *PLoS One*, 10(5): e0126282 Published online 2015 May 15. 10.1371/journal.pone.0126282 25978313 PMC4433213

[5] Sieghartsleitner R., Zuber C., Zibung M. & Conzelmann A. (2019). Science or Coaches' Eye?—Both! Beneficial Collaboration of Multidimensional Measurements and Coach Assessments for Efficient Talent Selection in Elite Youth Football. *J Sports Sci Med*., 18(1):32–43. 30787649 PMC6370964

[6] Vaeyens R., Lenoir M., Williams A.M. & Philippaerts R.M. (2008). Talent identification and development programmes in sport: current models and future directions. *Sports Med*., 38(9):703–14. 10.2165/00007256-200838090-00001 18712939

[7] Fransen J., Pion J., Vandendriessche J., Vandorpe B., Vaeyens R., Lenoir M., et al (2012). Differences in physical fitness and gross motor coordination in boys aged 6–12 years specializing in one versus sampling more than one sport. *J Sport Sci*., 30(4):379–386. 10.1080/02640414.2011.642808 22214429

[8] Fransen J., Deprez D., Pion J., Tallir I.B., D'Hondt E., Vaeyens R., et al (2014). Changes in Physical Fitness and Sports Participation Among Children With Different Levels of Motor Competence: A 2-Year Longitudinal Study. *Pediatr Exerc Sci*., 26(1):11–21. 10.1123/pes.2013-0005 24018944

[9] Balyi I., and Hamilton A. (2004). *Long-Term Athletic Development*: *Trainability in Childhood and Adolescence*. *Windows of Opportunity*. *Optimal Trainability*. Victoria: National Coaching Institute & British Columbia Advanced Training and Performance Institute.

[10] Brocherie F., Millet G.P., Hauser A., Steiner T., Wehrlin J.P., Rysman J., et al (2015). Association of Hematological Variables with Team-Sport Specific Fitness Performance. *PLoS One*. 10(12):e0144446 10.1371/journal.pone.0144446 eCollection 2015. . 26641647 PMC4671600

[11] Prommer N., Wachsmuth N., Thieme I., Wachsmuth C., Mancera Soto E. M., Hohmann A., and Schmidt W. F. (2018). Influence of endurance training during childhood on total hemoglobin mass. *Frontiers in Physiology*. 9, 251 10.3389/fphys.2018.00251 29618981 PMC5871736

[12] Astrand P.-O. (1952). *Experimental studies of physical working capacity in relation to sex and age*. Copenhagen: Ejnar Munksgaard.

[13] Schmidt W., and Prommer N. (2010). Impact of alterations in total hemoglobin mass on VO2max. *Exercise and Sport Science Review*. 38, 68–75. 10.1097/JES.0b013e3181d4957a 20335738

[14] Montero D., and Lundby C. (2017). Refuting the myth of non-response to exercise training: 'non-responders' do respond to higher dose of training. *Journal of Physiology*. 595, 3377–3387. 10.1113/JP273480 28133739 PMC5451738

[15] Hinrichs T., Franke J., Voss S., Bloch W., Schänzer W., and Platen P. (2010). *J Strength Cond Res*. 24(3):629–38. 10.1519/JSC.0b013e3181a5bc59 . 19704383

[16] Eastwood A., Bourdon P. C., Withers R., and Gore C. J. (2009). Longitudinal changes in hemoglobin mass and VO2max in adolescents. *European Journal of Applied Physiology*. 105, 715–721. 10.1007/s00421-008-0953-x 19084989

[17] Ulrich G., Bartsch P., and Friedmann-Bette B. (2011). Total haemoglobin mass and red blood cell profile in endurance-trained and non-endurance-trained adolescent athletes. Europ*ean Journal of Applied Physiology*, 111, 2855–2864. 10.1007/s00421-011-1920-5 21431423

[18] Garvican L. A., Martin D. T., McDonald W., and Gore C. J. (2010). Seasonal variation of haemoglobin mass in internationally competitive female road cyclists. *European Journal of Applied Physiology*. 109, 221–231. 10.1007/s00421-009-1349-2 20058020

[19] Silva D.A.S., de Lima T.R. & Tremblay M.S. (2018). Association between Resting Heart Rate and Health-Related Physical Fitness in Brazilian Adolescents. *Biomedical Research International*, 2018:3812197 Published online 2018 Jun 28. 10.1155/2018/3812197 30050928 PMC6046174

[20] Noriega-Sanchez S.A., Legaz-Arrese A., Suarez-Arrones L., Santalla A., Floria P. & Munguia-Izquierdo D. (2015). Forced inspiratory volume in the first second as predictor of front-crawl performance in young sprint swimmers. *Journal of Strength and Conditioning Research*, 29(1), 188–194. 10.1519/JSC.0000000000000634 . 25051007

[21] Doherty M. and Dimitriou L. (1997). Comparison of lung volume in Greek swimmers, land-based athletes, and sedentary controls using allometric scaling. *British Journal of Sports Medicine*. 31, 337–341. 10.1136/bjsm.31.4.337 9429014 PMC1332573

[22] Ghosh A. K., Ahuja A., and Khanna G. L. (1985). Pulmonary capacities of different groups of sportsmen in India. *British Journal of Sports Medicine*. 19, 232–234. 10.1136/bjsm.19.4.232 4092147 PMC1478382

[23] Bloomfield J., Blanksby B. A., Beard D. F., Ackland T. R., Badau A., and Elliott B. C. (1984). Biological characteristics of young swimmers, tennis players and non-competitors. *British Journal of Sports Medicine*. 18, 97–103. 10.1136/bjsm.18.2.97 6466937 PMC1859213

[24] Zhao K., Hohmann A. A., Chang Y., Zhang B., Pion J., and Gao B. (2019). Physiological, anthropometric, and motor characteristics of elite Chinese youth athletes from six different sports. *Frontiers in Physiology*. 10.3389/fphys.2019.00405 31105576 PMC6499036

[25] Lazovic B., Mazic S., Suzic-Lazic J., Djelic M., Djordjevic-Saranovic S., Durmic T., et al (2015). Respiratory adaptations in different types of sport. *European Review of Medicine and Pharmacological Science*. 19, 2269–2274. 26166653

[26] Hülsdünker T., Ostermann M. & Mierau A. (2019). The Speed of Neural Visual Motion Perception and Processing Determines the Visuomotor Reaction Time of Young Elite Table Tennis Athletes. *Frontiers in Behavioral Neuroscience*, *19*;13:165 10.3389/fnbeh.2019.00165 . PMCID: PMC6659573. eCollection 2019. 31379535 PMC6659573

[27] Nakamoto H. and Mori S. (2008). Sport-specific decision-making in a Go/NoGo reaction task: difference among nonathletes and baseball and basketball players. *Perceptual and Motor Skills*. 106, 163–70. 10.2466/pms.106.1.163-170 18459365

[28] Badau D., Baydil B., and Badau A. (2018). Differences among three measures of reaction time based on hand laterality in individual sports. *Sports* (Basel). 6, 45 10.3390/sports6020045 29910349 PMC6026828

[29] Chaouachi A., Brughelli M., Chamari K., Levin G. T., Ben-Abdelkrim N., Laurencelle L., et al (2009). Lower limb maximal dynamic strength and agility determinants in elite basketball players. *Journal of Strength and Conditioning Research*, 23(5), 1570–1577). 10.1519/JSC.0b013e3181a4e7f0 19620905

[30] Bunn J. A., Ryan G. A., Button G.R., and Zhang S. (2017). Evaluation of strength and conditioning measures with game success in Division I collegiate volleyball: A retrospective study. *Journal of Strength and Conditioning Research*, 2017, Aug 4, 10.1519/0000000000002181 (E-pub ahead of print). 28796124

[31] Tsaklis P., Malliaropoulos N., Mendiguchia J., Korakakis V., Tsapralis K., Pyne D., et al (2015). Muscle and intensity-based hamstring exercise classification in elite female track and field athletes: implications for exercise selection during rehabilitation. Open Access *Journal of Sports Medicine*. 26, 209–217. 10.2147/OAJSM.S79189 eCollection 2015. 26170726 PMC4492645

[32] Morouço P., Neiva H., González-Badillo J. J., Garrido N., Marinho D. A., and Marques M. C. (2011). Associations between dry land strength and power measurements with swimming performance in elite athletes: a pilot study. *Journal of Human Kinetics*. 29A:105–112. 10.2478/v10078-011-0065-2 Epub 2011 Oct 4. 23486734 PMC3588897

[33] Comfort P. and McMahon J.J. (2015). Reliability of maximal back squat and power clean performances in inexperienced athletes. *Journal of Strength and Conditioning Research*. 19, 3089–3096. 10.1519/JSC.0000000000000815 25559912

[34] Pollock S., Gaoua N., Johnston M.J., Cooke K., Girard O. & Mileva K.N. (2019). Training Regimes and Recovery Monitoring Practices of Elite British Swimmers. *Journal of Sports Science and Medicine*, 18(3), 577–585. eCollection 2019 Sep. . PMCID: PMC6683628. 31427881 PMC6683628

[35] Reis J.F., Alves F.B., Bruno P.M., Vleck V. & Millet G.P. (2012). Oxygen uptake kinetics and middle distance swimming performance. *Journal of Science and Medicine in Sport*, 15(1), 58–63. 10.1016/j.jsams.2011.05.012 Epub 2011 Jul 28. 21802360

[36] Phomsoupha M. & Laffaye G. (2015). The science of badminton: game characteristics, anthropometry, physiology, visual fitness and biomechanics. *Sports Medicine*, 45(4), 473–495. 10.1007/s40279-014-0287-2 . 25549780

[37] Rampichini S., Limonta E., Pugliese L., Cè E., Bisconti A.V., Gianfelici A., et al (2018). Heart rate and pulmonary oxygen uptake response in professional badminton players: comparison between on-court game simulation and laboratory exercise testing. *European Journal of Applied Physiology*, 118(11), 2339–2347. 10.1007/s00421-018-3960-6 Epub 2018 Aug 18. . 30121883

[38] Kondric M., Zagatto A.M. & Sekulic D. (2013). The physiological demands of table tennis: a review. Journa*l of Sports Science and Medicine*, 12(3), 362–370. eCollection 2013. . PMCID: PMC3772576. 24149139 PMC3772576

[39] Zagatto A.M., Morel E.A. & Gobatto C.A. (2010). Physiological responses and characteristics of table tennis matches determined in official tournaments. Jour*nal of Strength and Conditioning Research*, 24(4), 942–949. 10.1519/JSC.0b013e3181cb7003 . 20300034

[40] Zagatto A.M., Kondric M., Knechtle B., Nikolaidis P.T. & Sperlich B. (2018). Energetic demand and physical conditioning of table tennis players. A study review. *Journal of Sports Sciences*, 36(7), 724–731. 10.1080/02640414.2017.1335957 Epub 2017 Jun 5. . 28582628

[41] Berryman N., Mujika I., Arvisais D., Roubeix M., Binet C. Bosquet L. (2018). Strength Training for Middle- and Long-Distance Performance: A Meta-Analysis. *International Journal of Sports Physiology and Performance*, 13(1), 57–63. 10.1123/ijspp.2017-0032 . 28459360

[42] Ozmen T. & Aydogmus M. (2016). Effect of core strength training on dynamic balance and agility in adolescent badminton players. *Journal of Bodywork and Movement Therapy*, 20(3), 565–570. 10.1016/j.jbmt.2015.12.006 Epub 2015 Dec 19. . 27634079

[43] Zong X.N. & Li H. (2014). Physical growth of children and adolescents in China over the past 35 years. *Bull World Health Organ*., 92(8):555–564. 10.2471/BLT.13.126243 25177070 PMC4147404

[44] Wang H.J., Li Q., Guo Y., Song J.Y., Wang Z. & Ma J. (2017). Geographic variation in Chinese children' forced vital capacity and its association with long-term exposure to local PM10: a national cross-sectional study. *Environ Sci Pollut Res Int*., 24(28):22442–22449. 10.1007/s11356-017-9812-9 28803437

[45] Song Y., Wang H.J., Dong B., Wang Z., Ma J. & Agardh A. (2017).National Trends in Hemoglobin Concentration and Prevalence of Anemia among Chinese School-Aged Children, 1995–2010. *J Pediatr*., 183:164–169.e2. 10.1016/j.jpeds.2017.01.012 28153479

[46] Hutchison D.C.S., Barter C.E., and Martelli N.A. (1973). Errors in the measurement of vital capacity. A comparison of three methods in normal subjects and in patients with pulmonary emphysema *Thorax*. *1973 Sep;* 28(5): 584–587. PMCID: PMC470082. 10.1136/thx.28.5.584 . 4784379 PMC470082

[47] Jaggernath M., Naicker R., Madurai S., Brockman M. A., Ndung`u T., and Gelderbloom H. C. (2016). Diagnostic Accuracy of the HemoCue Hb 301, STAT-Site MHgb and URIT-12 Point-of-Care Hemoglobin Meters in a Central Laboratory and a Community Based Clinic in Durban, South Africa. *PLoS One*. *2016;* 11(4): e0152184 Published online 2016 Apr 5. 10.1371/journal.pone.0152184 PMCID: PMC4821624. . 27046200 PMC4821624

[48] Hawes M.R., and Martin A.D. (2001). Human Body Composition. In *Kinanthropometry and Exercise Physiology Laboratory Manual*: *Tests*, *Procedures and Data* (Vol I, pp. 00–00). London: Routledge.

[49] Stewart A., Marfell-Jones M., Olds T., and de Ridder H. (2011). *International standards for anthropometric assessment*. New Zeland: ISAK; LowerHutt.

[50] Hoffman J. R. (2006). *Norms for fitness*, *performance*, *and health*. Champaign, IL: Human Kinetics.

[51] Dorrell H. F., Moore J. M., Smith M. F., and Gee T. I. (2018). Validity and reliability of a linear positional transducer across commonly practised resistance training exercises. *Journal of Sports Sciences*, 10.1080/02640414.2018.1482588 29851551

[52] Miles J., and Shevlin M. (2001) App*lying Regression and Correlation*: *A Guide for Students and Researchers*. Sage:London.

[53] Moura T., Costa M., Oliveira S., Júnior M.B., Ritti-Dias R. & Santos M. (20199. Height and body composition determine arm propulsive force in youth swimmers independent of a maturation stage. *J Hum Kinet*., 42:277–284. 10.2478/hukin-2014-0081 25414760 PMC4234767

[54] Nikolić I., Nikolić I., Furjan-Mandić G. & Kondric M. (2014). The relationship of morphology and motor abilities to specific table tennis tasks in youngsters. *Coll Antropol*., 38(1):241–245. 24851624

[55] Handelsman D.J., Hirschberg A.L., and Bermon S. (2018). Circulating Testosterone as the Hormonal Basis of Sex Differences in Athletic Performance. *Endocr*. *Rev*. 2018 Oct 1;39(5):803–829. 10.1210/er.2018-00020 30010735 PMC6391653

[56] Coviello A.D., Kaplan B., Lakshman K.M., et al (2008). Effects of graded doses of testosterone on erythropoiesis in healthy young and older men. *J Clin Endocrinol Metab*. 93:914–919. 10.1210/jc.2007-1692 18160461 PMC2266950

[57] Hero M., Wickman S., Hanhijärvi R., Siimes M.A., and Dunkel L. (2005). Pubertal upregulation of erythropoiesis in boys is determined primarily by androgen. *J Pediatr*. 146(2):245–52. 10.1016/j.jpeds.2004.09.002 15689918

[58] Thomsen K., Riis B., Krabbe S., and Christiansen C. (1986). Testosterone regulates the haemoglobin concentration in male puberty. *Acta Paediatr Scand*. 75(5):793–6. 10.1111/j.1651-2227.1986.tb10292.x 3564947

[59] Steiner T., and Wehrlin J. P. (2011). Does hemoglobin mass increase from age 16 to 21 and 28 in elite endurance athletes? *Medicine and Science in Sports and Exercise*. 43, 1735–1743. 10.1249/MSS.0b013e3182118760 21311364

[60] Sitkowski D., Szygula Z., Pokrywka A., Turowski D., and Malczewska-Lenczowska J. (2018). Interrelationships between changes in erythropoietin, plasma volume, haemoglobin concentration, and total haemoglobin mass in endurance athletes. *Res Sports Med*. 26(3):381–389. 10.1080/15438627.2018.1447936 Epub 2018 Mar 8. 29516744

[61] Kamandulis S., Juodsnukis A., Stanislovaitiene J., Zuoziene I.J., Bogdelis A., Mickevicius M., et al (2020). Daily Resting Heart Rate Variability in Adolescent Swimmers during 11 Weeks of Training. *International Journal of Environmental Research and Public Health*, 17(6), 2097 Published online 2020 Mar 22. 10.3390/ijerph17062097 32235693 PMC7143004

[62] Mercier J., Vago P., Ramonatxo M., Bauer C., and Prefaut C. (1987). Effect of aerobic training quantity on the VO2 max of circumpubertal swimmers. *International Journal of Sports Medicine*. 8, 26–30. 10.1055/s-2008-1025635 3557779

[63] Lemaitre F., Coqart J.B., Chavallard F., Castres I., Mucci P., Costalat G., et al (2013). Effect of additional respiratory muscle endurance training in young well-trained swimmers. *Journal of Sport Science and Medicine*. 12, 630–638. eCollection 2013. PMC3873652 24421721

[64] Vasickova J., Neumannova K., and Svozil Z. (2017). The effect of respiratory muscle training on fin-swimmers' performance. *Journal of Sport Science and Medicine*. 16, 521–526. eCollection 2017 Dec. PMC5721182 29238252

[65] Simpson A.L., Ray A.D., Lundgren C.E., and Pendergast D.R. (2012). Energy cost of breathing at depth: effect of respiratory muscle training. *Undersea Hyperbaric Medicine*. 39, 829–834. 22908839

[66] Leetun D., Ireland M., and Wilson J. (2004). Core stability measures as risk factors for lower extremity injury in athletes. *Medicine and Science in Sports and Exercise*, 36: 926–934. 10.1249/01.mss.0000128145.75199.c3 15179160

[67] Buchheit M. (2014). Monitoring training status with HR measures: do all roads lead to Rome? *Frontiers in Physiology*, 5, 73 Published online 2014 Feb 27. 10.3389/fphys.2014.00073 PMCID: PMC3936188. . 24578692 PMC3936188

[68] Main L.C., Warmington S.A., Korn E. & Gastin P. (2016). Utility of the multi-component training distress scale to monitor swimmers during periods of training overload. *Research in Sports Medicine*, 24(3), 269–80. 10.1080/15438627.2016.1202828 Epub 2016 Jul 1. . 27368060

[69] McGuigan M. (2017). *Monitoring Training and Performance in Athletes*. Champaign, IL: Human Kinetics.

